# An Immersive Virtual Reality Exergame for People with Parkinson’s Disease

**DOI:** 10.1007/978-3-030-58796-3_18

**Published:** 2020-08-10

**Authors:** Weiqin Chen, Martin Bang, Daria Krivonos, Hanna Schimek, Arnau Naval

**Affiliations:** 8 Institute Integriert Studieren, JKU Linz, Linz, Austria; 9 Jack Baskin School of Engineering, UC Santa Cruz, Santa Cruz, CA USA; 10 Dipartimento di Meccanica, Politecnico di Milano, Milan, Italy; 11 Support Centre for Students with Special Needs, Masaryk University Brno, Brno, Czech Republic; Oslo Metropolitan University (OsloMet), POB 4 St. Olavs plass, 0130 Oslo, Norway

**Keywords:** Immersive virtual reality, Exergame, Parkinson’s disease

## Abstract

Parkinson’s disease is a neurodegenerative disorder that affects primarily motor system. Physical exercise is considered important for people with Parkinson’s disease (PD) to slow down disease progression and maintain abilities and quality of life. However, people with PD often experience barriers to exercises that causes low-level adherence to exercise plans and programs. Virtual Reality (VR) is an innovative and promising technology for motor and cognitive rehabilitation. Immersive VR exergames have potential advantages by allowing for individualized skill practice in a motivating interactive environment without distractions from outside events. This paper presents an immersive virtual reality (VR) exergame aiming at motor training on fingers and hand-and-eye coordination. The results from the usability study indicate that immersive VR exergames have potential to provide motivating and engaging physical exercise for people with PD. Through this research, we hope to contribute to evidence-based design principles for task-specific immersive VR exergames for patients with Parkinson’s Disease.

## Introduction

Parkinson’s disease (PD) is a neurodegenerative disorder affecting one in every 100 people over the age of 60 [1]. Apart from motor symptoms such as tremor and freezing of gait, cognitive symptoms in memory and executive function also cause challenges in daily living. PD affects primarily motor systems and exercises are found beneficial for people with PD in slowing down the decline and maintaining physical and cognitive abilities and quality of life [2]. However, people with PD often experience barriers to exercises which causes low level of adherence to exercise plans and programs [3]. Motivation is a critical factor for exercise adherence, which in turn is associated with important health benefits and improved quality of life [4]. Exergames which provide enjoyable exercise experiences and further increase the intrinsic motivation to adhere to the exercise programs are therefore becoming an increasingly popular alternative rehabilitation method for people with PD [5, 6].

Virtual Reality is an innovative and promising technology for motor and cognitive rehabilitation. A recent meta-analysis and systematic literature review conducted by Triegaardt et al. [7] on VR in rehabilitation of 1031 patients with PD found that VR training improved a number of outcomes in patients with PD including motor functioning, balance and coordination, cognitive function and quality of life. VR exergames have potential advantages by allowing for individualized skill practice in a motivating and engaging interactive environment. However, current VR exergames are mostly based on commercial game consoles such as Nintendo Wii, Sony PlayStation Eye and Microsoft’s Kinect. These games are often either too difficult for patients with PD or the games progress too quickly, failing to provide impairment-focused training or specifically address patients’ needs [8]. More specifically, these games are non-immersive VR, where the real physical world is enhanced by computer-generated digital information and users are not fully immersed in the virtual environment, therefore their experience can be interrupted. Immersive Virtual reality exergames, on the other hand, fully immerse users in the game, often with the help of a head mounted display (HMD) which allows users to focus entirely on the game without distractions from outside events.

This research aims to explore the potentials of fully immersive Virtual Reality exergames for people with PD. We have developed a simple VR exergame focusing on motor training on fingers and hand-and-eye coordination based on HTC vive and conducted user testing with patients with PD in a rehabilitation center to study the usability of the game.

## Related Work

Virtual Reality has been recognized as a promising tool for rehabilitation purposes by providing just-in-time feedback, allowing for repetitive practice, stimulating both cognitive and motor abilities simultaneously. Among others VR exergames have been used for rehabilitation of patients with cardiovascular disease [9] patients with spinal cord injury [10], and stroke patients [11].

For people living with PD, VR has mainly been used for gait and balance training [12, 13]. According to the recent systematic literature review [14] which examined eight trials with Virtual Reality therapy involving a total of 263 participants with PD, benefits of VR therapy include increased step and stride length and improvement in gait and balance. All the eight trials used non-immersive VR technology such Nintendo Wii.

In recent years with the increasing offers and decreasing price of immersive VR technology on the market, a few more studies have used immersive VR technology such as HTC vive and Oculus Rift for gait and balance training for people with PD [15, 16].

It is also suggested that Virtual Reality exergames hold some drawbacks for people with PD, such as cognitive overload and cyber sickness [5], and custom-made VR applications developed to offer disease-specific exercise may be able to overcome the drawbacks and prove to be more beneficial than commercial VR systems. However, there are very few custom-made VR immersive VR applications and most of the VR exergames are non-immersive and focus on training gait and balance. Even fewer VR applications are developed for training other aspects of motor systems than gait and balance for people with PD.

## Design and Development

The prototype design is based on an earlier project which focused on developing a mobile app for people with PD to carry on physical exercises using a human-centered design approach [17] where focus group interviews were conducted in a rehabilitation center with 20 people with PD and 7 health care workers. The design and implementation have taken an iterative process.

Related VR exergames and their design were studied in order to learn from their experiences. Design principles and considerations related to exergames and VR have been taken into considerations. For example, Burke et al. [18] identified two design principles for rehabilitation games: *meaningful play* where feedback and scoring mechanism are helpful for maintaining engagement, and *challenge* where different levels of difficulty are (dynamically) offered to target to the diversity of knowledge, skills, and needs of patients. Shaw et al. [19] discussed fiver major challenges in virtual reality exergame design: to overcome possible cyber sickness; to provide accurate motion control; to select appropriate view for the player; to address health and safety risks during high intensity exercises; and to address the issue of feedback latency.

People with PD often exhibit reduced manual dexterity that leads to difficulties in daily activities such as buttoning clothing, tying shoelaces, and handwriting. According to research by Agostino et al. [20], Parkinson’s disease impairs individual finger movements more than gross hand movements. Vanbellingen et al. [21] shows that an intensive, task specific home-based dexterity training program significantly improved fine motor skills in Parkinson’s disease and the effect generalized to dexterity-related activities of daily living (ADL) functions. Dockx et al. [14] recommended to study VR application in different disease stages to determine whether VR technology plays a role in the prevention of physical deterioration in early‐stage Parkinson’s disease and in the management of disease progression in the moderate to late stages.

The VR exergame prototype we have developed focuses on patients in early stage of PD and on exercise for hand-eye coordination and finger movements, in order to slow down the deterioration of motor performance in fingers. We aim to provide an easy to learn and use, motivating and enjoyable experience for the patients.

**Fig. 1.**
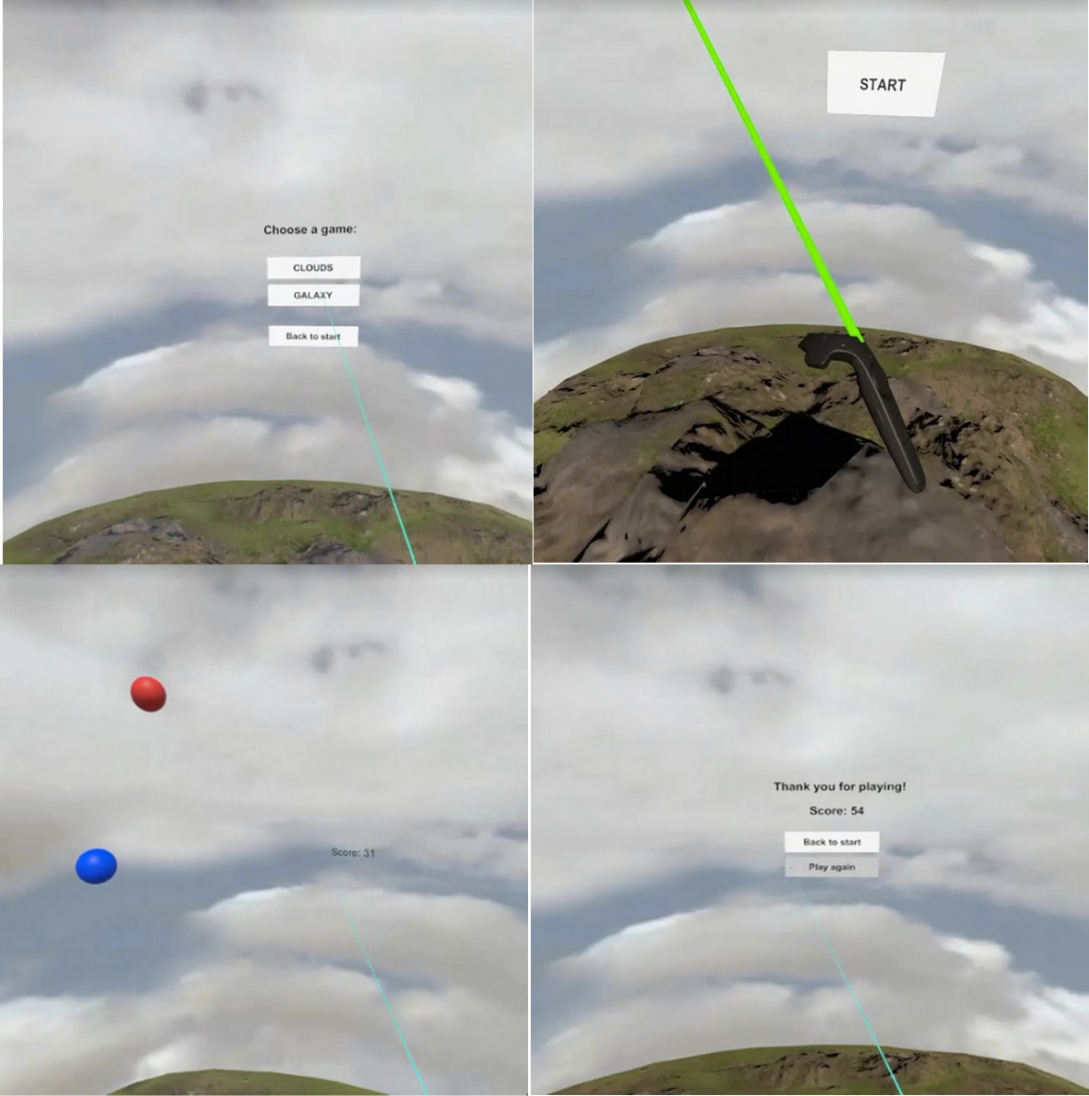
Screenshots of the first prototype

The exergame was developed on HTC vive and Unity was used as the game engine. Users wear the Head Mounted Display and use the controllers to play the game while sitting down to avoid sickness and keep safe. Feedback to users is provided visually and through the controllers. Both visual and haptic feedback are provided immediately without noticeable delay. The game consists of a simple interface where the user can choose the game setting in Cloud or Galaxy, as shown in Fig. 1. After that, the game shows balloons in different colors and background depending on the choice made by the user in the previous step. The user can move the controllers to target on the balloons and use finger to press the trigger and shoot. With each hit, a score will be shown. After the game is over, the user can see the total score.

## Evaluation

The user testing of the developed prototype was conducted at a rehabilitation center for patients with PD. Five early-stage PD patients participated in the testing. The participants consisted of two female and three male patients, with an age range 65–74 years old and an average of 5.6 years with PD. Participants rated themselves as ‘intermediate’ when asked about their computer knowledge and skills.

### Setting and Procedure

First, the purpose of the user testing and procedure was introduced and consent form was signed. After a pre-interview focusing on the demographic information, each participant was helped with putting on the Head Mounted Display and picked up the controllers. They were explained the basic of Virtual Reality and how the game should be played. They were then asked to play the game for two rounds (one in Cloud and one in Galaxy) and encouraged to think aloud and comment freely. After the two rounds, a post-interview was conducted to better understand their experience and gather feedback for improvement. Notes were taken during interviews as well as when the participants playing the game. Each participant was then asked to complete a System Usability Scale (SUS) questionnaire [22]. In SUS, levels of agreement with ten statements are scored using a five-point Likert scale from ‘strongly disagree’ to ‘strongly agree’. The average SUS score is 68.

### Data Analysis and Results

The participants were able to quickly understand the controls and the visual elements of the game including the trigger motion and laser select. The underlying game concept (shooting objects in a virtual environment) was well liked and participants reacted positively to the trigger mechanic. They found that the game was both fun and immersive, simple to use, and that they enjoyed its competitive nature. However, we have also identified some usability challenges. For example, the placement of the selection options was found too close to each other for participants with more intense hand tremors, making it difficult for them to select a given option with the handheld controller.

The results from the SUS questionnaire is shown in Table 1 where the scores from individual participant and individual question were calculated based on the choices made by the participants in the questionnaire. The mean SUS score is 90.

**Table 1.** Overview of the SUS scores.

Question (1-strongly disagree, 5-strongly agree)	Participant/Score					Mean
	P1	P2	P3	P4	P5	
1. I think that I would like to use this system frequently	2	2	2	4	3	2.6
2. I found the system unnecessarily complex	4	4	4	3	4	3.8
3. I thought the system was easy to use	4	4	4	4	3	3.8
4. I think that I would need the support of a technical person to be able to use this system	3	4	4	3	4	3.6
5. I found the various functions in this system were well integrated	3	4	4	4	4	3.8
6. I thought there was too much inconsistency in this system	1	4	3	3	4	3
7. I would imagine that most people would learn to use this system very quickly	4	4	4	4	4	4
8. I found the system very cumbersome to use	4	4	4	4	4	4
9. I felt very confident using the system	2	4	4	4	4	3.6
10. I needed to learn a lot of things before I could get going with this system	3	4	4	4	4	3.8

The participants have also made some suggestions regarding the game mechanics including different gameplay options such as size, moving speed, location, and type of the objects to hit, different scores for different types of objects, and their distances to the player in the virtual world. In addition, they thought that different backgrounds, settings, music and colors could make the game more engaging and immersive.

## Further Development

Based on the feedback from user testing of the first prototype, we have further developed the game with following improvements: level of difficulties; leader board for high scores; size, moving speed, location of balloons and their distances to the player; progress bar and number of streaks, and sound and animation.

**Fig. 2.**
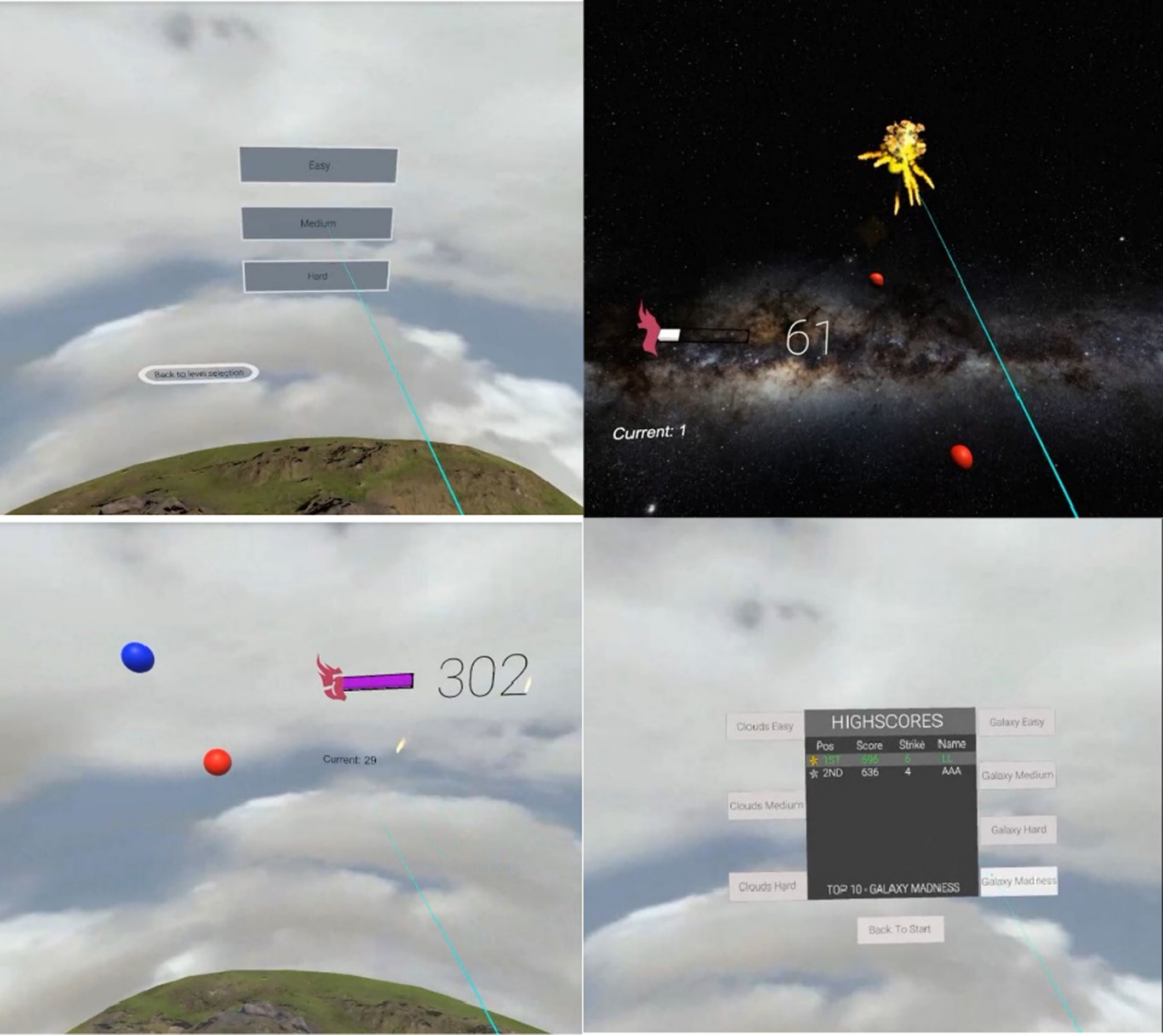
Screenshots of the second prototype

We have also addressed some general usability issues such as spaces between options so that PD patients with hand tremor do not choose options by mistakes. Figure 2 shows some screenshots of the improved prototype including the new features and functions based on user feedback.

## Conclusion and Future Work

In this paper, we have presented an immersive VR exergame prototype and the evaluation. The prototype was found user-friendly and it was well received by the participants. The high System Usability Scale scores indicated high levels of acceptability, ease of use, learnability and confidence when using the prototype. The small number of participants in the evaluation does not allow us to make any conclusions. However, the results indicate that immersive VR exergames is a promising tool for patients with PD. The evaluation also provides valuable input that guided further improvement of the prototype. More user testing with larger number of participants and longitudinal study are necessary in order to improve the usability of the game and understand its effects on improving hand-eye coordination and finger movements.

Through this research, we have found that although there are considerable research on exergames for rehabilitation in general and for people with PD specifically, there is a lack of evidence-based design guidelines for immersive VR and research on exergames with fully immersive VR for rehabilitation purpose is limited. Future research should therefore focus on design principles for task-specific immersive VR exergames for patients with Parkinson’s Disease taking into consideration the special needs of this user group.
